# Education and Self-Reported Health: Evidence from 23 Countries on the Role of Years of Schooling, Cognitive Skills and Social Capital

**DOI:** 10.1371/journal.pone.0149716

**Published:** 2016-02-22

**Authors:** Francesca Borgonovi, Artur Pokropek

**Affiliations:** 1 Department for Education and Skills, Organisation for Economic Co-operation and Development (OECD), Paris, France; 2 Institute of Philosophy and Sociology, Polish Academy of Science, Warsaw, Poland; Iowa State University, UNITED STATES

## Abstract

We examine the contribution of human capital to health in 23 countries worldwide using the OECD Survey of Adult Skills, a unique large-scale international assessment of 16–65 year olds that contains information about self-reported health, schooling, cognitive skills and indicators of interpersonal trust, which represents the cognitive dimension of social capital. We identify cross-national differences in education, skill and social capital gradients in self-reported health and explore the interaction between human capital and social capital to examine if and where social capital is a mediator or a moderator of years of schooling and cognitive abilities. We find large education gaps in self-reported health across all countries in our sample and a strong positive relationship between self-reported health and both literacy and trust in the majority of countries. Education and skill gradients in self-reported health appear to be largest in the United States and smallest in Italy, France, Sweden and Finland. On average around 5.5% of both the schooling gap in self-reported health and the literacy gap in self-reported health can be explained by the higher levels of interpersonal trust that better educated/more skilled individuals have, although the mediating role of trust varies considerably across countries. We find no evidence of a moderation effect: the relationships between health and years of schooling and health and cognitive skills are similar among individuals with different levels of trust.

## Introduction

Poor health is a major burden for the affected individual, but also for governments [1]. Recent estimates suggest that health expenditures account for as much as 9% of GDP across OECD countries; in the United States, they represent as much as 16% of GDP [2]. Moreover, there is a large body of evidence highlighting considerable disparities in health across population subgroups, with individuals with low socio-economic backgrounds and poor educational attainment being disproportionately more likely to be in ill health [3–7]. Tackling the high incidence of poor health and inequalities in health outcomes has risen to the top of policy agendas.

In recent years, new emphasis has been put on the social determinants of health [8]. The social context in which individuals live and the social connections they forge have profound effects on their health and well-being [9–18]. Individuals and groups interact with each other in ways that may influence their behaviors and lifestyles, their willingness and ability to take advantage of community resources, such as public services, and to cope with hardship and stress. Social characteristics add to the effects of material circumstances, from housing to transport, from working conditions to the quality of public services and institutions.

A large body of evidence both in the economics and public health literature documents a robust positive relationship between education and health (see [19–22] for reviews) and social capital and health [23]. Many definitions of social capital exist. In the context of this paper we borrow Francis Fukuyama’s definition of social capital. Fukuyama conceives social capital as *“shared norms or values that promote social cooperation*, *instantiated in actual social relationships”* [24].

With respect to the education-health gradient, challenges involved in assessing causality mean that there is no consensus on what determines observed relationships. Education and health may in fact interact in three not mutually exclusive ways: education may determine health, health may determine education and, finally, education and health may be jointly determined by another factor or set of factors [19, 25–28]. Two separate strands of literature have examined the education-health gradient: the first has attempted to establish the causality and the direction of the association using natural experiments that induced exogenous variations in educational attainment or participation. Empirical studies estimating the effect of education on health suggest that in some contexts exogenous increases in schooling are associated with better health outcomes [14, 29–34], while in others there is no effect [35–39]. The second strand has attempted to describe the mechanisms that may lead to the observed education-health link [3, 4, 40], whether this varies across countries [40–42], cohorts [43] and health indicators [44, 45].

One way in which education can promote health is by increasing labour market participation and the incomes individuals have. However, the education-health link is only partially explained by the increased income that highly educated individuals earn [46, 47]. Education might in fact promote better health through improved cognitive skills, for example by enabling individuals to be more efficient at maintaining good health [27], by prompting them to make better health choices [46, 48], and increasing their willingness and ability to access and use information [22] and by increasing their investment in social capital.

Interpersonal trust represents the cognitive dimension of social capital [49] and it has been found to be vital for well-being and economic prosperity [2, 50–54]. A large body of evidence also details a strong positive relationship between levels of interpersonal trust and health ([12, 55] for reviews). In our study we focus on interpersonal trust to examine social capital gradients in self-reported health. Interpersonal trust may promote better health by decreasing transaction costs, increasing access to material resources and to health-related information as well as promoting the development of informal insurance arrangements [56, 57]. When individuals trust anonymous others it is easier to reach a consensus out of different group interests, thus allowing for more efficient interactions [54, 58, 59]. Moreover, interpersonal trust provides sources of social and psychosocial support by establishing networks on which individuals can rely in times of need and by so doing may foster individual well-being [15, 60, 61].

We build on two lines of research: the first describes the relationship between education and health. The second describes the relationship between social capital and health. We investigate between country differences in education and skill gradients in health and explore if social capital is a mediator or moderator of the relationships between education, cognitive skills and health. Although the literature suggests that the health returns to schooling are heterogeneous, the specific sources of such heterogeneity are yet to be fully understood. Moreover, while the literature clearly indicates that education and social capital are associated with health outcomes, and that education is associated with higher levels of social capital, it is not clear if part of the education gradient can be explained by the higher levels of social capital that better educated individuals enjoy. Empirical studies of individual-level correlates of social capital have found that education is one of the most consistent predictors of social capital, at both the individual and area levels [62–68] and there is some evidence that such relationship is causal [69, 70].

Although our paper does not estimate causal effects, it provides new evidence and fills existing gaps in the literature by: *i)* detailing between-country differences in the size of the education-health link and the social capital-health link, *ii)* providing new insights on whether the education-health link varies when considering measures of schooling and measures of cognitive skills and *iii)* exploring if social capital is a mediator or a moderator of the relationships between education and health and skills and health.

We use a unique, cross-national dataset, the OECD Programme for the International Assessment of Adult Competencies (PIAAC). PIAAC contains a measure of self-reported health and educational attainment but also, and crucially for our study, cross-nationally comparable measures of cognitive skills and interpersonal trust.

The paper is structured as follows. We first examine the education, skills and trust gradients in self-reported health and whether these differ across countries. We then examine if the relationship between education/skills and health are mediated by levels of interpersonal trust (mediation analysis). Finally, we assess potential heterogeneous treatment effects (moderator analysis) to understand if the education and skills gradients in health are due to better-educated and better skilled individuals enjoying higher levels of interpersonal trust.

## Data and Methods

### Data

We use data from the OECD’s Survey of Adult Skills (PIAAC). Around 166 000 adults aged 16 to 65 were surveyed in the following 24 countries/national sub-regions: Australia, Austria, Belgium (Flanders), Canada, the Czech Republic, Cyprus, Denmark, Estonia, Finland, France, Germany, Ireland, Italy, Japan, Korea, the Netherlands, Norway, Poland, the Russian Federation, the Slovak Republic, Spain, Sweden, the United Kingdom (England and Northern Ireland), and the United States. Data collection took place from 1 August 2011 to 31 March 2012 in most participating countries, although in Canada data collection took place from November 2011 to June 2012, and in France it took place from September to November 2012. The target population for the survey was the non-institutionalized population, aged 16 to 65 years, residing in the country at the time of data collection, irrespective of nationality, citizenship or language status. The survey was administered in the official language or languages of each participating country and some countries gave respondents the possibility of participating in one of the widely spoken minority/regional languages (see [71] for technical details). In analyses presented in the paper we exclude individuals from the Russian Federation because of data concerns [71].

PIAAC has two main components: a background questionnaire and an assessment of literacy, numeracy and problem solving in technology-rich environments. The questionnaire was administered first in a CAPI (computer-assisted personal interviewing) format and response time ranged from 30 minutes to 45 minutes. Upon completion of the questionnaire, respondents sat a cognitive assessment, which took around one hour to complete. Depending on their computer skills, the assessment was delivered either on a laptop computer or as a fill-in paper booklet.

### Measurements

#### Outcome

Self-reported health is measured in PIAAC through the following statement: “How is your health these days?” to which respondents could answer on a five-point Likert scale ranging from “excellent”, “very good”, “good”, “fair” to “poor”. Self-reported health is an important predictor of mortality [72] and of the onset of disability and stress levels [73]. Self-reported health measures have high levels of validity and consistency, and the relationship between self-reported health and mortality does not vary by socio-economic group. Small differences can be observed, however, by gender and ethnic group [74, 75]. S1a Table shows the percentage of individuals who reported being in poor, fair, good, very good and excellent health in the countries that took part in the 2012 PIAAC study. On average, 4.5% of individuals in our sample reported being in poor health and 15% reported being in excellent health, while the majority reported being in either good or very good health (34% and 30%, respectively). Self-reported health varies considerably across countries. In Korea, over 10% of individuals between 25 and 65 years of age reported being in poor health, while in Cyprus less than 3% did. Similarly, in Estonia, Japan, Korea, Poland and the Slovak Republic less than 10% of individuals reported being in excellent health, while in Ireland, almost 25% of individuals did.

#### Education

We measure the education gradient in self-reported health through an indicator of the number of years an individual spent in school. This was derived by converting responses on education qualifications and mapping country-specific course length into years of schooling. S1a Table suggests that, on average, individuals in our sample attended 13 years of schooling. Individuals in Italy, Spain and France spent the least time in school (11 years), while those in Ireland and Australia spent the longest time (around 14.5 years), on average.

#### Cognitive ability

Cognitive ability is introduced using indicators of respondents’ literacy, measured through the standardized PIAAC literacy assessment. Achievement scores are based on IRT models: individuals’ response patterns to specific questions in their assessment are used to impute plausible value scores of achievement in the complete assessment. PIAAC estimates for each respondent and for each assessment domain a set of ten plausible values that can be used to assign to each respondent a probability estimate of their achievement on tasks at different levels of difficulty [71]. In the regression models, achievement measures are rescaled so that one unit is equal to 50 points, which roughly corresponds to the average standard deviation in our sample (46 score points).

S1a Table illustrates the extent to which individuals in different countries have different levels of literacy. Literacy scores are highest in Japan, Finland and the Netherlands, where adults score more than 280 points, on average, and lowest in Italy and Spain, where average literacy scores are below 260 points.

#### Interpersonal trust

We introduce an indicator of interpersonal trust measuring the extent to which individuals expect other members in their local communities to engage in cooperative behaviour and to hold shared social norms [50]. The measurement of interpersonal trust has a long tradition in social research, starting with the single item developed by Noelle-Neumann, “Generally speaking, do you believe that most people can be trusted or can’t you be too careful in dealing with people?” that was further developed by [76] into a three-item scale separating a “radius of trust” dimension and a “being careful/misanthropy” dimension [77]. While questions on interpersonal trust are widely used in comparative studies examining differences in trust across individuals and communities, some argue that the failure to specify in the question stem which “others” and which “people” are being referred to, may pose problems of comparability, particularly in cross-national research [21, 78–81].

PIACC contains two indicators of interpersonal trust: the statements “there are only a few people you can trust completely” and “if you are not careful, other people will take advantage of you”. Respondents could answer on a five-point Likert scale ranging from “strongly agree”, “agree”, “neither agree nor disagree”, “disagree” to “strongly disagree”. Both questions examine individuals’ beliefs about how they view others; but while the first is a pure indicator of how wide the radius of trust is, the second captures individuals’ expected behavioral responses and beliefs about their need to be careful in their relationships with others. In this paper, we use responses to the statement “there only a few people you can trust completely”.

S1a Table details levels of interpersonal trust. On average, about 24% of individuals reported strongly agreeing with the statement “there are only a few people you can trust completely”, in Italy 44% reported strongly agreeing with the statements, while in Denmark, Sweden and Finland around 10–11% of individuals and in Japan only 7% did.

#### Individual-level controls

Gender was reported by the respondent, and in all models we report the change in self-reported health that is associated with being a woman. We control for labor market participation (by introducing a dichotomous variable indicating if the respondent was unemployed or not in the labor force at the time of the survey) and occupation (by introducing a series of dichotomous variables to compare individuals who work in skilled occupations, semi-skilled white-collar occupations, semi-skilled blue-collar occupations and elementary occupations). We also control for respondents’ age (and model for non-linearities using a quadratic specification), the number of books individuals have in their home (to capture the availability of cultural resources), whether the respondent has children, whether the respondent lives with a partner and whether the respondent or the respondent’s parents, are foreign-born. S1b Table reports descriptive statistics for individual-level controls.

Because the aim of the paper is to examine the role education plays in promoting self-reported health, we exclude individuals who are still in their formative years (i.e. who are between the ages of 16 and 24). Across countries, on average, around 6% of respondents failed to report valid data on at least one of the variables used in our models (see S1a Table for detailed country-by-country descriptive statistics). In Japan and Germany less than 3% of respondents had missing information on key variables. However, in Cyprus, the Netherlands, Sweden and the United States over 15% of the overall sample had missing information on key variables. All our analyses are based on estimates obtained using multiple imputation of missing information [82] using ten imputation datasets, each generated with one literacy plausible value score. The imputation model included all the variables from the main analysis.

## Methods

We use regression analysis to examine the education, skill and interpersonal trust gradients in self-reported health status in a comparative perspective and assess the role of interpersonal trust as a potential mediator and/or moderator of education and skills gradients. We present estimates obtained using ordinary least squares in the body of the paper and present estimates obtained using ordered logistic regression models where we treat self-reported health and interpersonal trust as categorical variables in the S1–S4 Tables. Linear regression has at least three advantages over estimating effects using ordered logistic or similar models. First, cross country comparison in logistic regression models might be biased as coefficients depend both on effect sizes and the magnitude of unobserved heterogeneity [83, 84]. Second, the interpretation of results is easier than the interpretation of results from ordered logistic models for categorical variables. Finally, standard methods for testing mediation should not be used if either the mediator (in our case trust) or the outcome (in our case self-reported health) are categorical [85] and testing moderation (by means of interaction effects) is difficult in the presence of categorical independent variables with several categories (trust is coded using a 5 point Likert scale). While we cannot report results of statistical tests aimed at assessing mediation for ordered logistic models, all other results are not affected by the estimation procedure that we use.

S1b Table indicates that individuals in different countries differ markedly with respect to level of education attained and cognitive proficiency. In the presence of non-linearities in the associations between years of schooling/literacy and health, between-country comparisons would not be advisable. We examined potential non-linearities by introducing quadratic terms for years of schooling and literacy and found no evidence of non-linear relationships.

We fitted the following set of models:

M1a: *HEALTH* = *a*_1*a*_ + *β*_1*a*_*SCHOOLING*+*e*_1*a*_

M1b: *HEALTH* = *a*_1*b*_ + *β*_1*b*_*LITERACY*+*e*_1*b*_

M1c: *HEALTH* = *a*_1*c*_ + *β*_1*c*_*TRUST*+*e*_1*c*_

M2a: *HEALTH* = *a*_2_ + *β*_2_*SCHOOLING*+*β*_22_*LITERACY*+*e*_2_

M2b: $HEALTH=a_{3}+\beta_{3}SCHOOLING+\beta_{33}LITERACY+{v^{\prime }}_{3}^{}CONTROLS+e_{3}$

M3a: $HEALTH=a_{4}+\beta_{4}SCHOOLING+\beta_{44}LITERACY+\beta_{444}TRUST+{v^{\prime }}_{4}^{}CONTROLS+e_{4}$

M3b: $TRUST=a_{5}+\beta_{5}SCHOOLING+\beta_{55}LITERACY+{v^{\prime }}_{5}^{}CONTROLS+e_{5}$

Regression models presenting the education and skills gradients in self-reported health include the rich set of individual-level controls described earlier and account for missing data using multiple imputation. All continuous variables were standardized to have a mean of 0 and a standard deviation of 1 in the pooled sample.

We investigated mediation effects using Baron and Kenny’s approach [86], which compares estimates obtained in M2b, M3a and M3b:

Mediation effects occur when the main effects of literacy, schooling and trust from Model 3a are statistical significant and are larger than the main effects of literacy and schooling obtained in Model 2b. The Sobel test provides a test for the statistical significance of such difference [86], which can be represented as the percent of change in the model parameters and be interpreted as the proportion of the overall effects of schooling and literacy that are mediated by interpersonal trust.

Moderation effects were tested using interaction terms between interpersonal trust and years of schooling and literacy proficiency. Statistically significant interaction terms can be considered an indication of heterogeneous treatment effects. Positive interaction terms, if the main effects coefficients are also positive, can be interpreted as an indication that the schooling and literacy gradients are steeper among individuals with higher levels of interpersonal trust than among individuals with lower levels of interpersonal trust.

All point estimates were computed using sampling weights. Standard errors were calculated using the Jackknife replicate weights to consider the complex survey design of PIAAC, as well as the plausible values/multiple imputation methodology used to account for the fact that cognitive variables in PIAAC data are provided as random draws from posteriori distribution reflecting latent variables (see [71] for technical details) and that multiple imputation procedures were used.

## Results

The aim of this paper is to examine within-country disparities in the likelihood of individuals with different levels of schooling, literacy and interpersonal trust to report being in better or worse health and between-country disparities in the education, skill and trust gradients in self-reported health. We present results estimated using ordinary least squares in S2a and S3a Tables and report estimates obtained using ordered logistic regression in S2b and S3b Tables.

### The education and skill gradients in self-reported health

**Fig 1:**
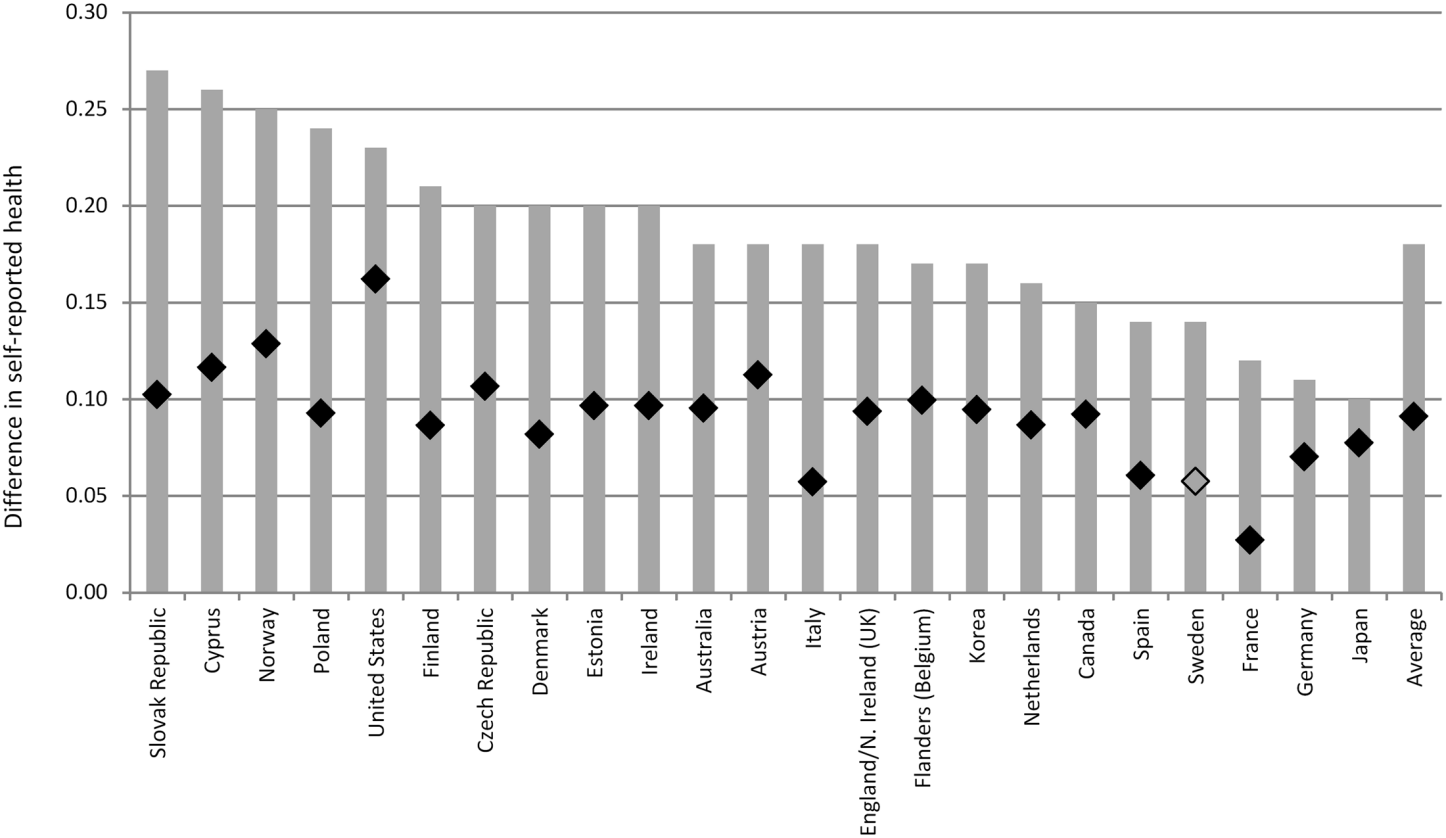
Cross-national differences in the schooling gradient in self-reported health. Grey bars report results from country-specific OLS regressions for model M2a (self-reported health regressed on years of schooling controlling for individual level literacy). The black diamond report estimates for model M3a (full set of controls included). Full results available in S1a and S2a Tables. Estimates that are not statistically significant at the 5% level are denoted in a lighter color.

**Fig 2:**
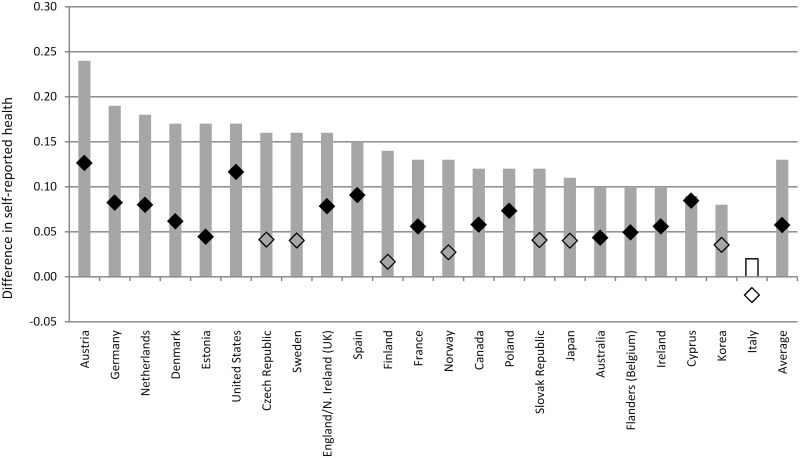
Cross-national differences in the literacy gradient in self-reported health. Grey bars report results from country-specific OLS regressions for model M2a (self-reported health regressed on literacy controlling for individual level years of schooling attended). The black diamond report estimates for model M3a (full set of controls included). Full results available in S1a and S2a Tables. Estimates that are not statistically significant at the 5% level are denoted in a lighter color.

Figs 1 and 2 illustrate the education and skill gradients in self-reported health in the countries in our sample. Across the 23 countries that participated in PIAAC, a difference in one standard deviation (s.d.) in the normalized years of schooling measure, which corresponds to around 3 years, is associated with a difference of 0.18 in the normalized self-reported health scale. Similarly, a difference of one standard deviation in literacy scores is associated with a difference of 0.13 in self-reported health. The strength of the association between years of schooling, literacy and self-reported health is reduced, but remains large and statistically significant, when we control for individuals’ background characteristics.

Results illustrated in Fig 1 suggest that the difference in self-reported health among individuals with similar background characteristics that is associated with a difference of one s.d. in years of schooling is large: 9% of a s.d. Similarly, Fig 2 indicates that the literacy gradient corresponds to 6% of a s.d. when controlling for background characteristics. The evidence presented is consistent with empirical studies showing that better-educated individuals are, on average, more likely to report better health than less-educated individuals, even after controlling for a variety of individual background characteristics [20, 47, 87].

Health disparities, by estimates of years of schooling and literacy levels, are very large, particularly considering that education and cognitive ability are closely associated and mutually reinforcing. Poorly educated individuals have lower levels of literacy, on average, than those who attended school for longer (5 additional years of schooling are associated with a 36-point higher score on the PIAAC proficiency scale, or around three quarters of a s.d.).

### The role of interpersonal trust

**Fig 3:**
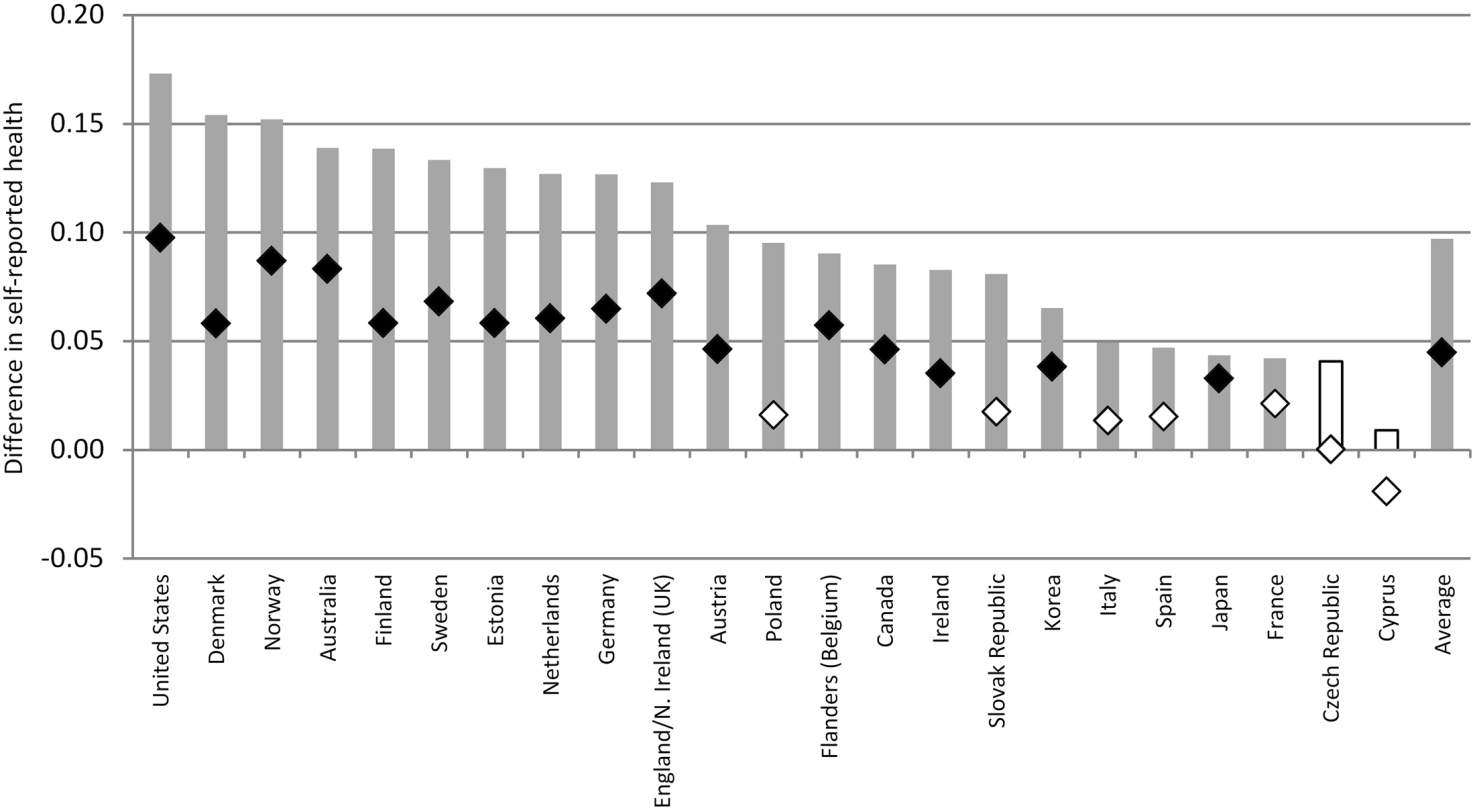
Cross-national differences in the trust gradient in self-reported health. Grey bars report results from country-specific OLS regressions of self-reported health regressed on trust. The black diamond report estimates for model M3a (full set of controls included). Estimates that are not statistically significant at the 5% level are denoted in a lighter color.

Consistent with past research and the hypotheses that we formulated, interpersonal trust is positively associated with self-reported health. Results presented in Fig 3 and S3a and S3b Table suggest that, on average, a difference of one s.d. in the normalized measure of interpersonal trust is associated with a difference of 10% of a s.d. in self-reported health. The interpersonal trust gradient in self-reported health remains statistically significant but is reduced when we control for years of schooling, skills and other key socio-economic and demographic characteristics of individuals: on average, across the countries that participated in the PIAAC study, the difference in self-reported health that is associated with a difference of one s.d. in the normalized trust variable among individuals that share similar background characteristics stands at 4% of a s.d.

### Between-country differences

We explore cross-national differences in the within-country relationship between human capital, social capital and self-reported health by estimating, for each country in our sample, the individual level relationship between years of schooling, literacy proficiency and interpersonal trust controlling for background characteristics.

Results presented in Figs 1 and 2 suggest that the education and skill gradients in self-reported health differ greatly across countries. When comparing individuals with similar socio-economic and demographic characteristics, the difference in self-reported health that is associated with schooling is largest in the United States (b = 0.162) and Norway (b = 0.129) and smallest in France, Italy and Sweden (in all these countries b<0.060). Similarly, the literacy gradient is over 0.10 in the United States and Austria, while no literacy gradient can be observed, after controlling for years of education as well as other individual characteristics, in Italy, Finland, Norway, South Korea, the Slovak Republic, and the Czech Republic.

**Fig 4:**
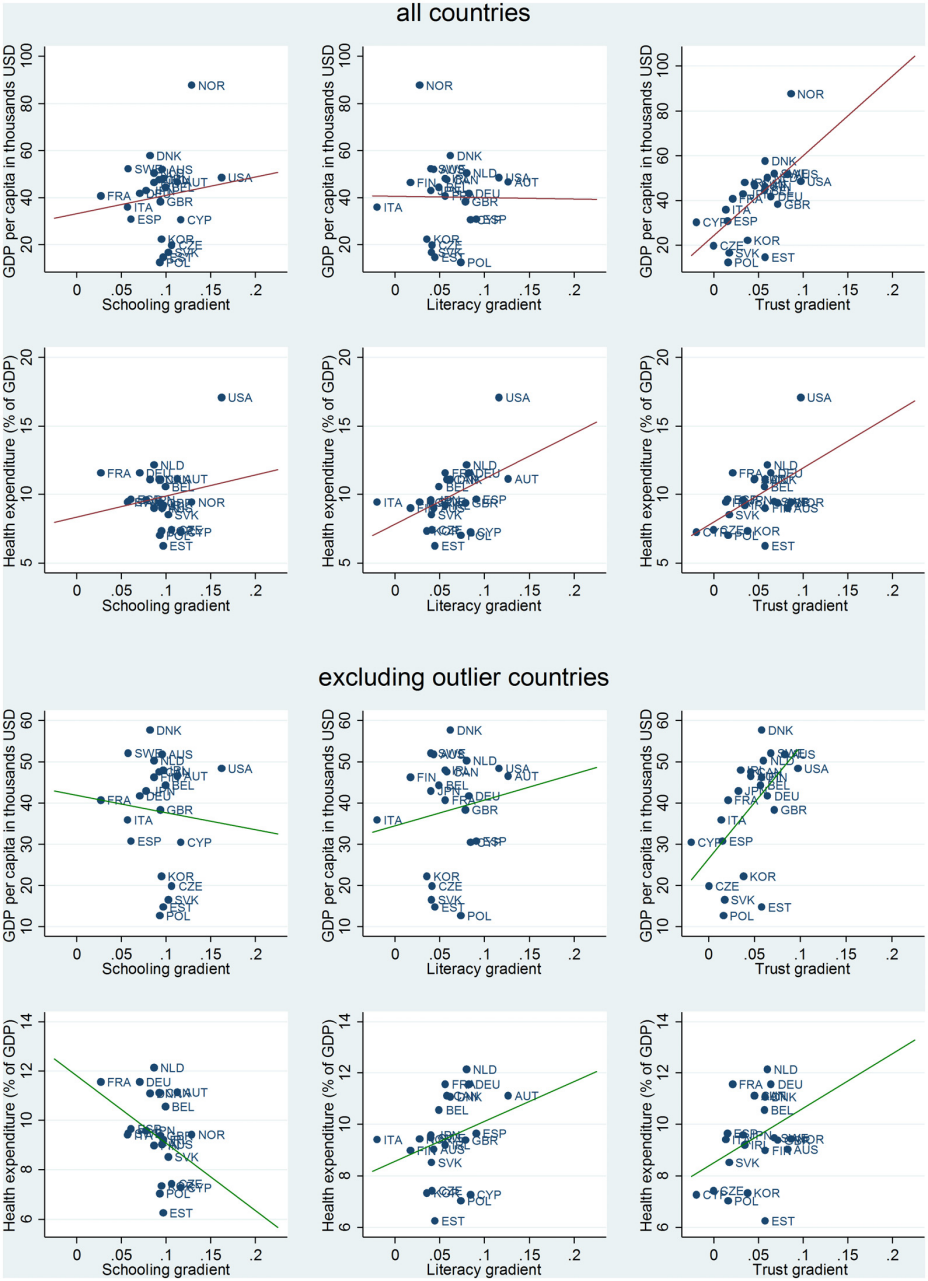
Between-country variations in health gradients. The red line represents associations on the entire sample (upper panel), the green line represents associations excluding outliers [lower panel, Norway (NOR) for GDP and the United States (USA) for health expenditures]

Just as the education and skills gradients in self-reported health differ greatly across countries, so do interpersonal trust gradients in self-reported health. After controlling for a rich set of individual level controls, individuals who report higher levels of interpersonal trust are considerably more likely to report being in better health in the United States, Norway and Australia (b_TRUST_>0.08 in these countries) while no difference in self-reported health between individuals with different levels of interpersonal trust could be found in Cyprus, the Czech Republic, Italy, Spain, Poland, the Slovak Republic and France. Fig 4 displays country level associations between GDP per capita, health expenditures and the schooling, literacy and trust gradients that we estimated in Model 3a. Results suggest that health and literacy gradients are not associated with level of economic development, as measured by GDP per capita. However, in countries with higher health expenditures as a percentage of GDP, literacy gradients tend to be steeper. Moreover, the relationship between interpersonal trust and health tends to be positive and larger in countries with higher GDP per capita and with higher health care expenditures.

We then turn to examining potential mediating and moderating effects of interpersonal trust. Do differences in interpersonal trust explain part of the relationships between self-reported health and years of schooling and health and literacy? And/or do we observe heterogeneous treatment effects of years or schooling and literacy proficiency across individuals who have accumulated different levels of interpersonal trust?

### The mediating and moderating role of interpersonal trust

We fit a series of country-specific models to test between-country differences in the mediating and moderating role of interpersonal trust and present results in S3a and S3b Table.

Results suggest that countries vary not only in the strength of the education and skill gradients in self-reported health, they also vary as to whether such disparities stem from an indirect mediating effect of interpersonal trust. The United States has the steepest health-schooling gradient and differences in levels of interpersonal trust in the population explain only around 6% of such gradient. On the other hand, Australia has a moderate health-schooling gradient and around 12% of such gradient is associated with the higher levels of interpersonal trust that better schooled individuals enjoy and the independent positive association that exists between interpersonal trust and health. In Cyprus, the health-schooling gradient and the health-skill gradients are large and interpersonal trust does not explain health differences associated with schooling and skills. In Austria and the United States, there is a large literacy gradient in self-reported health and only around 5% of such gradient can be explained by differences in interpersonal trust. On the other hand, in Australia, the skill gradient is small and over 15% of such gradient can be explained by the higher level of interpersonal trust that better skilled individuals enjoy.

S3a and S3b Table suggest that while in some countries levels of interpersonal trust play a mediating role of the relationship between years of schooling, literacy and health, in no country does trust play a moderating role of such relationships. We do not find any evidence of heterogeneous treatment effects of years of schooling and literacy levels across individuals with different levels of interpersonal trust in any of the countries in our sample.

## Conclusions and Discussion

Our paper builds on previous literature examining cross-national differences in education gradients in self-reported health. We use a new dataset containing measures for 23 countries of the number of years of schooling individuals attended, the literacy proficiency they demonstrate in an internationally agreed and validated assessment and their self-reported level of interpersonal trust to disentangle the independent relationship between educational attainment and cognitive skills and self-reported health and examine the extent to which human and social capital jointly explain within-country disparities in self-reported health status.

The first contribution of our paper is that it clearly illustrates that while individuals who have attended school for longer and who demonstrate better cognitive skills generally report being in better health than those who have attended school for less and who have lower literacy skills, education and skill gradients differ markedly across countries. In the United States, poorly educated individuals and individuals with low levels of literacy reported considerably worse health than individuals who attended school for longer or who have high levels of cognitive ability. By contrast, we cannot reject the hypothesis that there are no education and no skills gradients in self-reported health in Sweden.

In this study we report correlational evidence on the relationship between schooling, cognitive skills and self-reported health. Although no causal claims can be made on the basis of such evidence, the between-country variations in the strength of the associations between years of schooling and health and literacy and health support the notion that health production functions are context dependent. The amount of resources invested in health care provision, the organization of health care systems, levels of economic development and the level of social cohesion that different communities enjoy may determine not only the overall health and well-being of populations, but may also have important distributional implications. By shaping access to resources and the cognitive demands individuals face when they engage in lifestyle choices and when they interact with health care providers, all these factors may lead to better or worse outcomes for those individuals who are able to acquire better resources or to navigate complexity. An important finding of the paper is that the years of schooling and skills gradients are associated but are both conceptually and empirically distinct. In some countries both the education and the skills gradients are large/small. However, in other countries the education gradient is large and the literacy gradient is small and *vice versa*. This suggests that in some countries access to resources may be at the root of disparities in health, while in others disparities may be more associated with complexity and the (in)ability to effectively acquire and process information.

In countries with higher health expenditures as a percentage of GDP, literacy gradients tend to be steeper. This relationship may have to do with the different organizational features and funding arrangements of countries with greater health expenditures. The health and social welfare systems of some countries, such as the United States, require individuals to make choices that can determine their health outcomes, such as selecting a health insurance plan or a healthcare provider. While these systems may grant greater choice and freedom to individuals, they also require individuals to have the skills that are necessary to effectively acquire, process and react to information. Health care in systems that lack universal health care provision and where coverage is offered in an open market, is a literacy demanding space [88]. In such systems it may be more difficult to serve effectively the health needs that individuals with poor cognitive skills have. Recent evidence suggests that in the United States poor literacy and numeracy skills are an independent risk factors for poor health across different socio-demographic groups [88]. Moreover, several studies indicate that cognitive skills can have significant effects on lifestyles and health behaviors [46]. We find that literacy is, on average, less strongly associated with self-reported health in national healthcare systems (data on health systems characteristics was taken from the 2012 OECD Health System Characteristics Survey 2012).

Reducing education and skill disparities in health, particularly in systems that have no universal provision of health care can be achieved through very different policy responses: by providing better skills and education to all, by modifying the system to provide basic universal health care to all, or by using regulation and taxation policies to limit the consumption of unhealthy products and engagement in unhealthy behaviors. While some individuals may lose a certain degree of choice if such measures are adopted, societies may ultimately gain, as individuals with poor cognitive skills may enjoy better health outcomes.

The fact that an education gradient exists in virtually all countries even when controlling for cognitive ability suggests that a crucial mechanism that determines education disparities in self-reported health is related to social stratification and the unequal availability and quality of health and social services. The steepness of the education gradient, after controlling for any differences in the cognitive ability of individuals who attended school for a different number of years, is a clear indicator of how much social status and social class give some individuals unequal access to goods and services, such as good primary and specialist care, short waiting lists for key procedures, or a home in a safe neighborhood with good air quality and outside spaces in which to exercise. It can also signal differences in the employment opportunities individuals with different levels of education enjoy in different countries, and other factors, such as work-related stress and sense of agency, that can negatively affect the health of individuals with low social status.

Finally, we identify a positive relationship between interpersonal trust (representing an important dimension of social capital) and health in many countries. Some policy makers and scholars view nurturing social capital as a potentially useful tool for promoting good health, but others have warned that such an agenda may exacerbate social inequalities in health, and result in a culture that blames individuals for their illnesses and diseases [89–91]. Our results suggest that social capital may be one of the pathways through which the education and the skill gradients in self-reported health express themselves. On the other hand we do not find any support for the notion that years of schooling or literacy proficiency are more positively associated with self-reported health among individuals with high levels of social capital.

## Supporting Information

S1 Table Descriptive statistics of core variables. (PDF)

S2 Table Gradients for health. (PDF)

S3 Table The mediating and moderating role of social capital. (PDF)

S4 Table Country level characteristics. (PDF)
