# Supplementary material for: Education and Self-Reported Health: Evidence from 23 Countries on the Role of Years of Schooling, Cognitive Skills and Social Capital
Source: PLoS One. 2016 Feb 22;11(2):e0149716. doi: 10.1371/journal.pone.0149716 (PMC4763098; doi:10.1371/journal.pone.0149716)
Supplement: S1 Table — (PDF) [file pone.0149716.s001.pdf]

Table S1a Descriptive statistics of core variables

Country	Final sample size	% of sample with missing info	Health					There are only a few people you can trust completely					Schooling		Literacy	
			Poor	Fair	Good	Very good	Excellent	Strongly agree	Agree	Neither agree nor disagree	Disagree	Strongly disagree				
	n	%	%	%	%	%	%	%	%	%	%	%	mean	sd	mean	sd
Australia	6,272	4.6%	4.27	11.70	31.71	35.06	17.26	22.84	47.87	14.44	13.06	1.79	14.52	2.66	280.89	50.31
Austria	4,030	4.8%	3.86	14.65	28.85	33.44	19.19	35.29	33.91	12.78	15.61	2.40	12.08	2.62	268.03	43.95
Canada	21,265	3.6%	3.04	8.23	29.23	37.30	22.20	22.08	46.27	15.69	13.66	2.30	13.59	2.69	274.06	50.36
Cyprus	3,675	15.4%	2.37	12.90	29.07	37.22	18.44	41.28	52.62	4.22	1.45	0.43	12.48	3.30	269.42	40.72
Czech Republic	4,485	3.0%	3.32	9.59	45.01	28.79	13.29	24.08	62.13	8.48	4.35	0.97	13.26	2.58	272.83	40.8
Denmark	6,101	2.5%	4.82	13.89	21.05	40.16	20.09	10.11	29.51	19.15	32.78	8.46	12.95	2.63	270.33	48.13
Estonia	4,467	2.2%	6.75	32.74	38.11	16.09	6.32	25.67	50.09	14.49	8.70	1.04	12.32	2.71	274.1	44.37
Finland	3,929	12.1%	3.03	17.00	39.08	26.82	14.07	11.17	29.47	20.14	32.99	6.23	12.63	3.11	287.47	49.47
Flanders (Belgium)	5,456	7.4%	2.97	12.41	41.20	29.39	14.03	17.83	46.08	16.88	17.21	2.00	12.60	2.92	274.12	47.66
France	4,204	4.4%	4.55	16.40	37.66	25.79	15.59	38.13	31.55	16.01	11.45	2.86	11.32	3.71	260.62	49.12
Germany	5,144	1.6%	3.99	8.62	25.31	43.36	18.72	28.54	37.22	26.05	7.21	0.98	13.43	2.63	268.85	47.1
Ireland	3,993	2.5%	3.17	9.60	26.44	36.69	24.10	27.40	47.90	11.25	11.71	1.75	14.62	3.35	266.09	48.03
Italy	4,037	10.1%	5.36	14.93	35.90	30.60	13.21	44.09	38.10	10.52	5.15	2.13	10.61	4.01	248.62	44.76
Japan	5,516	1.5%	5.88	22.08	45.32	20.30	6.42	7.19	25.43	35.08	25.59	6.72	13.22	2.35	296.75	39.9
Korea	4,141	3.2%	10.40	43.47	33.07	9.57	3.50	22.80	51.81	14.89	9.18	1.32	12.85	3.31	268.62	41.93
Netherlands	3,502	15.6%	3.35	16.33	38.49	24.72	17.12	12.11	43.04	19.18	23.10	2.58	13.45	2.70	282.34	49.17
Norway	4,767	2.6%	4.80	14.32	31.30	33.41	16.16	18.31	35.38	14.74	22.54	9.03	14.38	2.52	279.24	47.55
Poland	4,455	2.8%	5.38	16.71	47.89	25.26	4.76	23.14	62.53	8.30	4.99	1.03	12.78	3.10	264.58	48.18
Slovak Republic	4,848	4.1%	5.40	17.00	38.68	30.09	8.84	30.99	54.86	5.53	7.68	0.95	13.26	2.78	274.11	39.63
Spain	3,519	3.0%	4.50	18.81	40.31	23.70	12.68	31.16	37.38	14.04	14.08	3.34	11.40	3.65	250.61	49.34
Sweden	6,281	18.0%	3.90	13.08	29.66	30.96	22.40	10.84	25.90	19.74	33.93	9.58	12.34	2.57	279.6	50.4
England/N. Ireland (UK)	6,049	3.6%	4.25	9.70	26.89	37.15	22.02	24.18	48.10	13.63	12.16	1.93	13.15	2.30	279.58	46.36
United States	3,503	16.1%	4.31	12.23	28.82	32.09	22.54	30.49	45.67	12.23	8.66	2.95	13.69	3.05	269.06	51.33
**Average**	123,639	6.44%	4.51	15.93	34.31	29.91	15.35	24.34	42.73	15.11	14.66	3.16	12.91	2.92	272.17	46.46

Table S1b Descriptive statistics of control variables

Country	Number of books at home						Occupational classification of respondent's job					Age		Female	Children	Living with partner	1st & 2nd gen. immigrants	Employed
	10 books or less	11 to 25 books	26 to 100 books	101 to 200 books	201 to 500 books	More than 500 books	Skilled	Semi-skilled white-collar	Semi-skilled blue-collar	Elementary	Not working in the 5 years prior to the survey							
	%	%	%	%	%	%	%	%	%	%	%	mean	sd	%	%	%	%	%
Australia	12.95	12.62	35.26	18.07	13.46	7.64	42.33	21.66	17.11	7.49	11.40	43.95	11.62	50.32	72.31	70.54	39.54	75.57
Austria	15.15	18.25	33.51	14.84	11.44	6.80	37.65	24.19	19.94	8.09	10.14	44.69	11.24	50.64	74.23	73.86	19.95	75.81
Canada	17.07	16.23	33.83	15.60	11.84	5.42	51.14	19.86	15.58	5.31	8.11	44.93	11.53	50.23	73.81	72.72	33.55	79.13
Cyprus	33.23	22.23	28.94	8.74	4.42	2.44	33.79	30.73	14.47	6.32	14.69	43.98	11.76	54.04	79.15	78.93	10.80	71.41
Czech Republic	1.94	6.10	33.25	26.71	21.93	10.07	32.78	21.60	29.19	6.53	9.89	44.6	11.85	49.91	79.91	70.35	6.61	72.53
Denmark	8.55	11.73	28.67	20.76	18.98	11.32	45.05	22.00	17.39	7.81	7.74	45.55	11.5	49.99	76.90	72.33	11.38	77.37
Estonia	4.21	9.23	27.82	23.26	22.32	13.17	41.00	16.34	26.44	8.01	8.20	44.47	11.74	52.96	81.59	71.57	26.16	78.03
Finland	9.15	13.82	34.10	18.50	16.45	7.99	39.67	24.24	21.55	6.12	8.43	45.93	11.94	49.89	74.06	71.57	4.02	76.39
Flanders (Belgium)	25.84	20.50	29.73	11.61	8.66	3.66	42.67	21.30	16.12	7.40	12.51	45.9	11.45	49.47	79.17	80.23	8.40	77.01
France	21.76	16.91	31.62	14.17	9.46	6.09	36.44	22.11	20.11	9.63	11.72	45.12	11.63	51.37	77.76	71.91	17.90	70.81
Germany	11.58	14.60	34.35	17.60	14.20	7.67	35.75	26.74	20.85	7.38	9.29	45.16	11.06	49.49	73.26	73.09	20.17	79.61
Ireland	22.92	18.42	31.51	12.87	9.79	4.49	32.08	25.92	19.65	7.32	15.03	42.95	11.32	51.39	71.16	69.16	18.20	66.02
Italy	32.53	22.61	26.00	10.80	5.29	2.77	24.26	22.08	21.85	9.06	22.75	44.96	11.42	51.48	67.07	67.98	7.52	61.38
Japan	17.80	20.33	36.67	12.36	8.69	4.15	32.46	32.31	18.71	4.83	11.69	45.28	11.64	48.86	69.37	70.80	0.18	81.16
Korea	22.90	18.98	35.19	12.72	7.12	3.08	24.96	31.94	19.15	9.23	14.71	44.01	10.91	49.57	76.87	69.38	1.16	74.45
Netherlands	15.86	15.22	29.09	16.40	14.98	8.46	49.25	22.90	10.05	5.77	12.03	45.31	11.43	50.07	72.55	75.28	14.94	78.13
Norway	5.94	8.70	29.31	22.23	19.76	14.06	47.62	26.22	14.85	3.76	7.56	44.66	11.55	50.11	79.06	73.37	14.06	81.60
Poland	15.82	20.10	34.59	15.20	10.17	4.12	31.25	17.33	26.26	6.57	18.58	44.04	12.05	51.10	74.45	72.39	1.36	67.32
Slovak Republic	10.46	18.02	38.35	19.06	10.43	3.67	35.03	18.47	24.79	6.86	14.84	43.9	11.75	50.33	77.48	72.09	2.67	68.38
Spain	21.45	20.84	32.65	11.85	8.72	4.48	26.44	27.83	19.02	13.25	13.46	44.14	11.18	50.06	69.98	72.63	11.98	62.90
Sweden	7.48	8.39	29.44	20.64	20.92	13.13	44.47	24.49	19.06	4.73	7.24	45.4	11.81	49.91	76.21	71.18	20.04	81.00
England/N. Ireland (UK)	11.73	15.17	33.76	16.95	14.25	8.14	39.95	29.22	14.34	7.38	9.10	43.83	11.43	50.33	72.14	68.29	19.19	77.79
United States	20.24	18.44	31.28	15.54	9.36	5.15	45.49	23.16	15.17	6.77	9.40	44.61	11.54	51.91	76.90	69.09	19.36	76.75
**Average**	15.52	15.89	32.21	16.63	12.83	6.92	38.02	23.80	19.50	7.06	11.61	44.54	11.51	50.64	75.16	71.97	13.94	74.37
