# Supplementary material for: Education and Self-Reported Health: Evidence from 23 Countries on the Role of Years of Schooling, Cognitive Skills and Social Capital
Source: PLoS One. 2016 Feb 22;11(2):e0149716. doi: 10.1371/journal.pone.0149716 (PMC4763098; doi:10.1371/journal.pone.0149716)
Supplement: S2 Table — (PDF) [file pone.0149716.s002.pdf]

Table S2a OLS gradients for health

Country	Model 1			Model 2a						Model 2b (with controls)					
	Years of schooling			Years of schooling			Literacy			Years of schooling			Literacy		
	Coef.		(S.E.)	Coef.		(S.E.)	Coef.		(S.E.)	Coef.		(S.E.)	Coef.		(S.E.)
Australia	0.24	***	(0.02)	0.18	***	(0.02)	0.10	***	(0.02)	0.11	***	(0.03)	0.05	**	(0.02)
Austria	0.30	***	(0.02)	0.18	***	(0.02)	0.24	***	(0.02)	0.12	***	(0.02)	0.13	***	(0.02)
Canada	0.21	***	(0.01)	0.15	***	(0.01)	0.12	***	(0.01)	0.10	***	(0.01)	0.06	***	(0.02)
Cyprus	0.28	***	(0.02)	0.26	***	(0.02)	0.09	***	(0.03)	0.12	***	(0.02)	0.08	***	(0.03)
Czech Republic	0.27	***	(0.02)	0.20	***	(0.02)	0.16	***	(0.03)	0.11	***	(0.02)	0.04		(0.03)
Denmark	0.29	***	(0.02)	0.20	***	(0.02)	0.17	***	(0.02)	0.09	***	(0.02)	0.07	***	(0.02)
England/N. Ireland (UK)	0.26	***	(0.02)	0.18	***	(0.03)	0.16	***	(0.02)	0.10	***	(0.03)	0.08	***	(0.02)
Estonia	0.28	***	(0.01)	0.20	***	(0.01)	0.17	***	(0.02)	0.10	***	(0.01)	0.05	**	(0.02)
Finland	0.28	***	(0.02)	0.21	***	(0.02)	0.14	***	(0.02)	0.10	***	(0.02)	0.02		(0.02)
Flanders (Belgium)	0.23	***	(0.02)	0.17	***	(0.02)	0.10	***	(0.02)	0.11	***	(0.02)	0.05	*	(0.02)
France	0.18	***	(0.01)	0.12	***	(0.01)	0.13	***	(0.02)	0.03	*	(0.01)	0.06	***	(0.02)
Germany	0.22	***	(0.02)	0.11	***	(0.02)	0.19	***	(0.02)	0.08	**	(0.03)	0.09	***	(0.02)
Ireland	0.25	***	(0.02)	0.20	***	(0.02)	0.10	***	(0.02)	0.10	***	(0.02)	0.06	**	(0.02)
Italy	0.19	***	(0.01)	0.18	***	(0.02)	0.02		(0.02)	0.06	**	(0.02)	-0.02		(0.02)
Japan	0.15	***	(0.02)	0.10	***	(0.02)	0.11	***	(0.02)	0.08	***	(0.02)	0.04		(0.03)
Korea	0.21	***	(0.01)	0.17	***	(0.01)	0.08	***	(0.02)	0.10	***	(0.02)	0.03		(0.02)
Netherlands	0.28	***	(0.02)	0.16	***	(0.02)	0.18	***	(0.02)	0.10	***	(0.02)	0.09	***	(0.02)
Norway	0.32	***	(0.02)	0.25	***	(0.02)	0.13	***	(0.02)	0.14	***	(0.02)	0.04		(0.02)
Poland	0.30	***	(0.01)	0.24	***	(0.02)	0.12	***	(0.02)	0.09	***	(0.02)	0.07	***	(0.02)
Slovak Republic	0.32	***	(0.01)	0.27	***	(0.02)	0.12	***	(0.02)	0.10	***	(0.02)	0.04		(0.02)
Spain	0.21	***	(0.01)	0.14	***	(0.01)	0.15	***	(0.02)	0.06	***	(0.02)	0.09	***	(0.02)
Sweden	0.23	***	(0.02)	0.14	***	(0.02)	0.16	***	(0.02)	0.06	*	(0.03)	0.05		(0.02)
United States	0.34	***	(0.02)	0.23	***	(0.02)	0.17	***	(0.02)	0.17	***	(0.03)	0.12	***	(0.02)
**Average**	0.25	***	(0.02)	0.18	***	(0.02)	0.13	***	(0.02)	0.10	***	(0.02)	0.06	**	(0.02)

Controls: age, age^2, gender, employment, number of books at home, immigrant status, occupational classification of respondent's job, having children.

*** $p<0.001$; ** $p<0.01$; * $p<0.05$

Table S2b ordered logit gradients for health

Country	Model 1			Model 2a						Model 2b (with controls)					
	Years of schooling			Years of schooling			Literacy			Years of schooling			Literacy		
	Coef.		(S.E.)	Coef.		(S.E.)	Coef.		(S.E.)	Coef.		(S.E.)	Coef.		(S.E.)
Australia	0.44	***	(0.04)	0.34	***	(0.04)	0.19	***	(0.04)	0.21	***	(0.05)	0.09	*	(0.04)
Austria	0.53	***	(0.03)	0.33	***	(0.03)	0.46	***	(0.04)	0.23	***	(0.05)	0.27	***	(0.05)
Canada	0.40	***	(0.02)	0.28	***	(0.03)	0.22	***	(0.03)	0.19	***	(0.03)	0.12	***	(0.03)
Cyprus	0.56	***	(0.04)	0.51	***	(0.04)	0.18	***	(0.05)	0.25	***	(0.05)	0.19	**	(0.06)
Czech Republic	0.60	***	(0.04)	0.46	***	(0.05)	0.33	***	(0.07)	0.28	***	(0.05)	0.10		(0.07)
Denmark	0.51	***	(0.03)	0.36	***	(0.04)	0.28	***	(0.03)	0.17	***	(0.05)	0.12	**	(0.04)
England/N. Ireland (UK)	0.45	***	(0.04)	0.33	***	(0.05)	0.26	***	(0.04)	0.20	***	(0.06)	0.15	***	(0.05)
Estonia	0.59	***	(0.03)	0.44	***	(0.03)	0.37	***	(0.04)	0.26	***	(0.04)	0.12	**	(0.04)
Finland	0.57	***	(0.03)	0.43	***	(0.04)	0.29	***	(0.04)	0.21	***	(0.04)	0.04		(0.04)
Flanders (Belgium)	0.48	***	(0.04)	0.36	***	(0.05)	0.20	***	(0.04)	0.25	***	(0.05)	0.10	*	(0.04)
France	0.34	***	(0.02)	0.22	***	(0.02)	0.25	***	(0.03)	0.06	*	(0.03)	0.12	***	(0.03)
Germany	0.42	***	(0.04)	0.23	***	(0.05)	0.35	***	(0.04)	0.16	**	(0.06)	0.16	***	(0.05)
Ireland	0.46	***	(0.03)	0.37	***	(0.03)	0.20	***	(0.04)	0.20	***	(0.04)	0.12	**	(0.04)
Italy	0.35	***	(0.03)	0.33	***	(0.03)	0.05		(0.05)	0.12	*	(0.04)	-0.05		(0.05)
Japan	0.32	***	(0.04)	0.21	***	(0.05)	0.22	***	(0.05)	0.18	***	(0.05)	0.09		(0.05)
Korea	0.49	***	(0.02)	0.41	***	(0.03)	0.20	***	(0.05)	0.25	***	(0.04)	0.10	*	(0.05)
Netherlands	0.54	***	(0.04)	0.32	***	(0.04)	0.35	***	(0.04)	0.21	***	(0.05)	0.17	***	(0.05)
Norway	0.58	***	(0.04)	0.47	***	(0.04)	0.24	***	(0.04)	0.27	***	(0.05)	0.09	*	(0.04)
Poland	0.71	***	(0.03)	0.58	***	(0.04)	0.27	***	(0.04)	0.26	***	(0.05)	0.21	***	(0.04)
Slovak Republic	0.65	***	(0.03)	0.55	***	(0.04)	0.26	***	(0.05)	0.26	***	(0.05)	0.11	*	(0.05)
Spain	0.40	***	(0.02)	0.26	***	(0.03)	0.29	***	(0.03)	0.13	***	(0.04)	0.20	***	(0.04)
Sweden	0.39	***	(0.04)	0.23	***	(0.04)	0.28	***	(0.04)	0.10	*	(0.06)	0.09		(0.05)
United States	0.64	***	(0.04)	0.44	***	(0.05)	0.33	***	(0.05)	0.35	***	(0.05)	0.25	***	(0.05)
**Average**	0.50	***	(0.03)	0.37	***	(0.04)	0.26	***	(0.04)	0.21	***	(0.05)	0.13	**	(0.05)

Controls: age, age^2, gender, employment, number of books at home, immigrant status, occupational classification of respondent's job, having children.
*** p<0.001; ** p<0.01; * p<0.05
