# Supplementary material for: Education and Self-Reported Health: Evidence from 23 Countries on the Role of Years of Schooling, Cognitive Skills and Social Capital
Source: PLoS One. 2016 Feb 22;11(2):e0149716. doi: 10.1371/journal.pone.0149716 (PMC4763098; doi:10.1371/journal.pone.0149716)
Supplement: S3 Table — (PDF) [file pone.0149716.s003.pdf]

Table S3a The mediating and moderating role of interpersonal trust (OLS)

Country	Model 3a - Trust regressed on: (with controls)				Model 3b - Health regressed on: (with controls)						Test for mediating effects of trust				Model 3c - Moderating effects (with controls)			
	schooling		literacy		schooling		literacy		trust		schooling		literacy		schooling		literacy	
	Coef.	(S.E.)	Coef.	(S.E.)	Coef.	S.E.	Coef.	S.E.	Coef.	S.E.	% change	p-value	% change	p-value	Coef.	S.E.	Coef.	S.E.
Australia	0.15***	(0.02)	0.10***	(0.02)	0.10***	(0.03)	0.04*	(0.02)	0.08***	(0.01)	11.84%	0.00	16.33%	0.00	-0.03	(0.02)	0.02	(0.02)
Austria	0.10***	(0.02)	0.12***	(0.02)	0.11***	(0.02)	0.13***	(0.02)	0.05**	(0.02)	3.81%	0.03	4.13%	0.02	-0.02	(0.01)	0.01	(0.02)
Canada	0.12***	(0.02)	0.06***	(0.02)	0.09***	(0.01)	0.06***	(0.02)	0.05***	(0.01)	5.52%	0.00	4.87%	0.00	0.01	(0.01)	-0.01	(0.01)
Cyprus	0.06***	(0.02)	-0.02	(0.02)	0.12***	(0.02)	0.08***	(0.03)	-0.02	(0.03)	-1.11%	0.32	0.45%	0.34	-0.04	(0.02)	0.03	(0.04)
Czech Republic	0.08***	(0.03)	0.07**	(0.03)	0.11***	(0.02)	0.04	(0.03)	0.00	(0.02)	0.03%	0.40	0.04%	0.40	0.00	(0.04)	0.00	(0.04)
Denmark	0.18***	(0.02)	0.12***	(0.02)	0.08***	(0.02)	0.06*	(0.02)	0.06***	(0.02)	11.35%	0.00	9.92%	0.00	0.00	(0.02)	0.01	(0.02)
England/N. Ireland (UK)	0.15***	(0.03)	0.09***	(0.03)	0.09***	(0.03)	0.08***	(0.02)	0.07***	(0.02)	10.18%	0.00	7.23%	0.01	-0.03	(0.03)	0.00	(0.02)
Estonia	0.08***	(0.02)	0.03**	(0.01)	0.10***	(0.01)	0.04*	(0.02)	0.06***	(0.01)	4.69%	0.00	3.73%	0.06	0.02	(0.02)	0.02	(0.02)
Finland	0.15***	(0.02)	0.01	(0.03)	0.09***	(0.02)	0.02	(0.02)	0.06***	(0.01)	9.31%	0.00	3.45%	0.37	-0.01	(0.02)	0.02	(0.01)
Flanders (Belgium)	0.16***	(0.02)	0.03	(0.02)	0.10***	(0.02)	0.05*	(0.02)	0.06***	(0.02)	8.66%	0.00	3.24%	0.16	0.02	(0.02)	0.02	(0.02)
France	0.07***	(0.01)	0.03*	(0.01)	0.03*	(0.01)	0.06***	(0.02)	0.02	(0.01)	5.26%	0.11	1.03%	0.19	0.00	(0.01)	-0.01	(0.02)
Germany	0.09***	(0.03)	0.08***	(0.02)	0.07**	(0.03)	0.08***	(0.02)	0.06***	(0.02)	7.69%	0.02	5.75%	0.01	-0.03	(0.02)	0.00	(0.02)
Ireland	0.11***	(0.02)	0.02	(0.02)	0.10***	(0.02)	0.06*	(0.02)	0.04*	(0.02)	3.77%	0.05	1.46%	0.24	0.00	(0.02)	0.01	(0.02)
Italy	0.08***	(0.02)	0.06**	(0.02)	0.06**	(0.02)	-0.02	(0.02)	0.01	(0.02)	1.82%	0.29	-3.82%	0.30	-0.02	(0.02)	0.00	(0.02)
Japan	0.15***	(0.02)	-0.01	(0.02)	0.08***	(0.02)	0.04	(0.03)	0.03*	(0.02)	5.98%	0.05	-1.10%	0.34	0.02	(0.03)	0.02	(0.02)
Korea	0.08***	(0.02)	-0.04*	(0.02)	0.09***	(0.02)	0.04	(0.02)	0.04**	(0.01)	3.13%	0.02	-4.17%	0.12	-0.01	(0.02)	0.01	(0.02)
Netherlands	0.18***	(0.02)	0.10***	(0.03)	0.09***	(0.02)	0.08***	(0.02)	0.06***	(0.01)	11.24%	0.00	6.88%	0.01	-0.01	(0.02)	0.00	(0.02)
Norway	0.15***	(0.03)	0.15***	(0.02)	0.13***	(0.02)	0.03	(0.02)	0.09***	(0.01)	8.94%	0.00	31.92%	0.00	0.00	(0.02)	-0.02	(0.02)
Poland	0.03	(0.02)	0.04**	(0.02)	0.09***	(0.02)	0.07***	(0.02)	0.02	(0.02)	0.59%	0.30	0.87%	0.28	0.00	(0.02)	-0.01	(0.02)
Slovak Republic	0.05**	(0.02)	-0.01	(0.02)	0.10***	(0.02)	0.04	(0.02)	0.02	(0.02)	0.83%	0.26	-0.31%	0.38	-0.01	(0.02)	0.03	(0.02)
Spain	0.11***	(0.02)	0.01	(0.02)	0.06***	(0.02)	0.09***	(0.02)	0.02	(0.01)	2.77%	0.23	0.24%	0.34	0.01	(0.01)	0.00	(0.02)
Sweden	0.09***	(0.03)	0.09***	(0.02)	0.06	(0.03)	0.04	(0.02)	0.07***	(0.02)	10.06%	0.01	13.14%	0.01	-0.02	(0.02)	0.02	(0.02)
United States	0.11***	(0.03)	0.07**	(0.03)	0.16***	(0.03)	0.12***	(0.02)	0.10***	(0.02)	6.02%	0.00	5.42%	0.03	0.01	(0.02)	-0.02	(0.02)
**Average**	0.11***	(0.02)	0.05**	(0.02)	0.09***	(0.02)	0.06*	(0.02)	0.04**	(0.02)	5.81%	0.02	5.14%	0.08	-0.01	(0.02)	0.01	(0.02)

Controls: age, age^2, gender, employment, number of books at home, immigrant status, occupational classification of respondent's job, having children.

*** p<0.001; ** p<0.01; * p<0.05

Table S3b The mediating and moderating role of interpersonal trust

	Model 3a - Trust regressed on:						Model 3b - Health regressed on: (with controls)										Test for mediating effects of trust		Model 3c - Moderating effects (with controls)							
	schooling			literacy			schooling			literacy			trust				schooling	literacy	schooling				literacy			
	Coef.		(S.E.)	Coef.		(S.E.)	Coef.		S.E.	Coef.		S.E.	t=2 Coef.	t=3 Coef.	t=4 Coef.	t=5 Coef.	% change	% change	t=2 Coef.	t=3 Coef.	t=4 Coef.	t=5 Coef.	t=2 Coef.	t=3 Coef.	t=4 Coef.	t=5 Coef.
Australia	0.30	***	(0.04)	0.18	***	(0.03)	0.19	***	(0.05)	0.08	*	(0.04)	0.05	0.09	**0.35**	**0.70**	9.4%	16.4%	0.03	-0.17	-0.16	0.13	-0.09	0.09	0.05	0.09
Austria	0.15	***	(0.04)	0.23	***	(0.04)	0.22	***	(0.05)	0.26	***	(0.05)	0.07	0.06	**0.22**	**0.38**	3.0%	3.5%	-0.13	-0.13	-0.08	-0.11	0.08	-0.04	0.02	0.16
Canada	0.22	***	(0.03)	0.11	***	(0.03)	0.18	***	(0.03)	0.11	***	(0.03)	0.01	0.07	**0.20**	**0.34**	4.6%	5.3%	-0.09	0.07	-0.06	0.23	-0.06	-0.10	-0.08	-0.04
Cyprus	0.17	**	(0.06)	-0.02		(0.06)	0.25	***	(0.05)	0.19	**	(0.06)	-0.06	-0.06	-0.17	-0.21	-1.6%	0.3%	-0.10	-0.23	-0.16	-0.41	0.07	0.10	0.03	0.55
Czech Republic	0.19	**	(0.07)	0.26	***	(0.08)	0.28	***	(0.06)	0.09		(0.07)	-0.05	0.02	0.05	-0.08	1.3%	2.0%	0.05	0.04	-0.23	-0.11	-0.10	-0.05	-0.04	0.42
Denmark	0.33	***	(0.04)	0.20	***	(0.04)	0.15	**	(0.05)	0.11	**	(0.04)	-0.11	-0.09	0.10	**0.41**	12.3%	9.8%	0.05	-0.04	-0.07	0.04	-0.11	0.01	-0.03	-0.05
England/N. Ireland (UK)	0.28	***	(0.05)	0.17	***	(0.05)	0.18	***	(0.06)	0.14	*	(0.05)	**0.17**	**0.29**	**0.43**	0.27	8.9%	9.4%	-0.09	0.02	**-0.29**	0.19	-0.10	-0.12	0.00	-0.12
Estonia	0.19	***	(0.04)	0.10	***	(0.03)	0.25	***	(0.04)	0.12	**	(0.04)	**0.16**	0.13	**0.41**	**0.52**	3.8%	3.5%	0.07	-0.07	0.12	0.28	-0.02	0.19	0.06	-0.18
Finland	0.26	***	(0.04)	0.03		(0.04)	0.20	***	(0.04)	0.04		(0.04)	0.08	0.04	**0.21**	**0.74**	6.8%	-9.7%	0.08	0.12	-0.09	0.03	-0.02	-0.05	0.13	0.02
Flanders (Belgium)	0.33	***	(0.04)	0.07		(0.04)	0.23	***	(0.05)	0.10	*	(0.04)	0.07	0.13	**0.33**	0.31	8.2%	1.7%	0.00	0.16	0.09	0.09	0.04	0.17	0.06	0.24
France	0.15	***	(0.02)	0.04		(0.03)	0.05	*	(0.03)	0.12	***	(0.03)	-0.01	-0.02	0.15	0.18	6.4%	1.1%	**-0.15**	-0.15	0.01	0.18	0.01	-0.04	-0.05	-0.22
Germany	0.18	***	(0.05)	0.17	***	(0.04)	0.15	**	(0.06)	0.15	**	(0.05)	0.10	**0.24**	**0.34**	**0.61**	7.2%	5.5%	-0.05	-0.22	-0.20	-0.19	-0.02	-0.03	0.04	0.04
Ireland	0.24	***	(0.04)	0.04		(0.04)	0.19	***	(0.04)	0.12	**	(0.04)	0.01	0.09	0.18	0.23	3.4%	1.1%	-0.14	-0.03	0.04	-0.07	0.01	-0.06	-0.01	0.22
Italy	0.14	***	(0.04)	0.15	**	(0.05)	0.11	**	(0.04)	-0.05		(0.05)	-0.01	0.15	0.08	0.02	3.7%	-0.7%	-0.07	**-0.20**	-0.13	0.12	0.01	-0.11	0.04	-0.06
Japan	0.31	***	(0.05)	-0.01		(0.05)	0.17	***	(0.05)	0.09		(0.05)	-0.13	**-0.31**	0.17	**0.41**	6.9%	-2.7%	-0.02	0.06	0.16	-0.19	0.03	0.04	0.02	0.21
Korea	0.18	***	(0.04)	-0.09		(0.05)	0.24	***	(0.04)	0.11	*	(0.05)	0.04	0.05	0.20	**0.69**	2.4%	-2.3%	-0.16	**-0.22**	-0.09	-0.01	**0.24**	**0.29**	0.12	0.08
Netherlands	0.34	***	(0.05)	0.18	***	(0.05)	0.19	***	(0.05)	0.15	***	(0.05)	-0.05	-0.01	0.16	**0.58**	9.6%	6.3%	0.13	-0.10	-0.08	0.06	-0.14	-0.01	-0.07	-0.02
Norway	0.24	***	(0.05)	0.22	***	(0.04)	0.25	***	(0.05)	0.07		(0.05)	0.15	**0.25**	**0.38**	**0.66**	9.2%	24.5%	0.06	-0.12	0.01	0.04	-0.01	-0.12	-0.14	-0.06
Poland	0.07		(0.06)	0.11	*	(0.05)	0.26	***	(0.05)	0.21	***	(0.04)	-0.07	-0.15	0.12	0.23	1.1%	0.5%	-0.08	-0.03	-0.14	0.07	-0.13	-0.18	-0.21	0.07
Slovak Republic	0.11	*	(0.05)	-0.03		(0.06)	0.25	***	(0.05)	0.11	*	(0.05)	0.05	0.15	0.20	-0.32	1.0%	0.0%	0.03	-0.10	-0.05	0.09	-0.02	0.15	0.08	0.26
Spain	0.19	***	(0.03)	0.04		(0.04)	0.13	***	(0.04)	0.20	***	(0.04)	**-0.15**	0.01	0.02	0.15	3.8%	-1.3%	0.01	0.04	0.03	0.02	-0.15	-0.06	-0.09	0.00
Sweden	0.17	***	(0.05)	0.14	**	(0.04)	0.09		(0.06)	0.08		(0.05)	-0.04	-0.08	0.19	**0.51**	9.7%	7.6%	-0.16	-0.16	-0.22	-0.08	0.01	-0.05	0.12	0.06
United States	0.17	***	(0.05)	0.16	**	(0.05)	0.33	***	(0.05)	0.24	***	(0.05)	0.07	**0.27**	**0.44**	**0.78**	4.5%	4.1%	-0.01	-0.16	-0.03	0.31	0.00	0.06	-0.13	-0.28
**Average**	0.21	***	(0.05)	0.11	***	(0.05)	0.20	***	(0.05)	0.12	***	(0.05)	0.02	0.06	**0.21**	**0.35**	5.1%	4.0%	-0.03	-0.07	-0.07	0.03	-0.02	0.00	0.00	0.06

Controls: age, age^2, gender, employment, number of books at home, immigrant status, occupational classification of respondent's job, having children.

*** p<0.001; ** p<0.01; * p<0.05

Table S3c The mediating and moderating role of interpersonal trust (OLS) – effects of tertiary education

	Model 3a - Trust regressed on: (with controls)						Model 3b - Health regressed on: (with controls)									Test for mediating effects of trust				Model 3c - Moderating effects (with controls)			
	Tertiary education			literacy			Tertiary education			literacy			trust			Tertiary education		literacy		Tertiary education		literacy	
	Coef.		(S.E.)	Coef.		(S.E.)	Coef.		S.E.	Coef.		S.E.	Coef.		S.E.	% change	p-value	% change	p-value	Coef.	S.E.	Coef.	S.E.
Australia	0.15	***	(0.03)	0.12	***	(0.02)	0.07		(0.04)	0.05	**	(0.02)	0.09	***	(0.01)	16.08%	0.00	16.15%	0.00	-0.03	(0.03)	0.02	(0.02)
Austria	0.02		(0.05)	0.13	***	(0.02)	0.19	***	(0.04)	0.14	***	(0.02)	0.05	**	(0.02)	0.64%	0.36	4.64%	0.01	-0.06	(0.04)	0.01	(0.02)
Canada	0.08		(0.04)	0.09	***	(0.02)	0.16	***	(0.04)	0.07	***	(0.02)	0.05	***	(0.01)	2.29%	0.07	6.18%	0.00	-0.01	(0.04)	0.00	(0.01)
Cyprus	0.06		(0.04)	0.00		(0.02)	0.18	***	(0.05)	0.10	***	(0.02)	-0.01		(0.03)	-0.42%	0.36	0.07%	0.40	-0.11	(0.07)	0.03	(0.03)
Czech Republic	-0.04		(0.04)	0.09	***	(0.02)	0.22	**	(0.08)	0.05		(0.03)	0.01		(0.02)	-0.11%	0.38	1.07%	0.38	0.13	(0.13)	0.00	(0.03)
Denmark	0.18	***	(0.04)	0.14	***	(0.02)	0.18	***	(0.04)	0.06	**	(0.02)	0.06	***	(0.01)	5.48%	0.01	11.73%	0.00	-0.02	(0.04)	0.02	(0.02)
England/N. Ireland (UK)	0.13	**	(0.04)	0.10	***	(0.03)	0.10		(0.05)	0.08	***	(0.02)	0.08	***	(0.02)	9.01%	0.01	7.98%	0.01	0.01	(0.05)	-0.01	(0.02)
Estonia	0.00		(0.04)	0.04	***	(0.01)	0.09	**	(0.03)	0.06	**	(0.02)	0.06	***	(0.01)	-0.15%	0.40	4.72%	0.01	0.02	(0.04)	0.02	(0.02)
Finland	0.19	***	(0.05)	0.03		(0.02)	0.10	*	(0.04)	0.03		(0.02)	0.06	***	(0.01)	10.95%	0.01	6.88%	0.17	0.04	(0.04)	0.02	(0.01)
Flanders (Belgium)	0.10	*	(0.04)	0.07	***	(0.02)	0.10	*	(0.04)	0.07	***	(0.02)	0.07	***	(0.01)	6.57%	0.04	6.29%	0.01	0.00	(0.04)	0.03	(0.02)
France	0.04		(0.03)	0.05	***	(0.01)	0.05		(0.03)	0.06	***	(0.02)	0.02		(0.01)	1.84%	0.22	1.81%	0.11	0.02	(0.04)	-0.02	(0.02)
Germany	0.05		(0.06)	0.09	***	(0.02)	0.07		(0.07)	0.10	***	(0.02)	0.07	***	(0.02)	4.07%	0.29	6.15%	0.01	-0.03	(0.07)	-0.01	(0.02)
Ireland	0.14	***	(0.04)	0.04	*	(0.02)	0.15	***	(0.04)	0.07	***	(0.02)	0.04	*	(0.02)	3.48%	0.05	2.40%	0.12	0.01	(0.04)	0.01	(0.02)
Italy	0.15	***	(0.04)	0.06	**	(0.02)	0.05		(0.04)	-0.01		(0.02)	0.02		(0.02)	4.31%	0.26	-11.59%	0.26	-0.03	(0.05)	0.00	(0.02)
Japan	0.06		(0.05)	0.02		(0.02)	0.16	**	(0.05)	0.04		(0.02)	0.04	*	(0.02)	1.29%	0.23	1.33%	0.30	0.01	(0.07)	0.03	(0.02)
Korea	0.05		(0.03)	-0.02		(0.02)	0.21	***	(0.04)	0.04	*	(0.02)	0.04	**	(0.01)	0.95%	0.18	-1.56%	0.29	0.01	(0.04)	0.01	(0.02)
Netherlands	0.20	***	(0.04)	0.13	***	(0.02)	0.09	*	(0.04)	0.09	***	(0.02)	0.06	***	(0.01)	12.31%	0.00	8.10%	0.00	0.01	(0.04)	0.00	(0.02)
Norway	0.15	*	(0.06)	0.16	***	(0.03)	0.20	***	(0.05)	0.04		(0.02)	0.09	***	(0.01)	6.49%	0.02	27.65%	0.00	-0.01	(0.03)	-0.01	(0.02)
Poland	-0.02		(0.04)	0.05	*	(0.02)	0.17	***	(0.04)	0.08	***	(0.02)	0.02		(0.02)	-0.23%	0.35	1.00%	0.26	0.04	(0.05)	-0.02	(0.02)
Slovak Republic	0.02		(0.04)	0.00		(0.02)	0.15	**	(0.05)	0.05	*	(0.02)	0.02		(0.02)	0.28%	0.36	0.08%	0.40	0.06	(0.06)	0.01	(0.02)
Spain	0.12	**	(0.04)	0.04		(0.02)	0.05		(0.04)	0.10	***	(0.02)	0.02		(0.01)	4.11%	0.18	0.70%	0.21	0.06	(0.03)	-0.01	(0.02)
Sweden	0.04		(0.06)	0.11	***	(0.03)	0.19	**	(0.06)	0.04		(0.02)	0.07	***	(0.01)	1.56%	0.30	16.77%	0.00	0.00	(0.04)	0.02	(0.02)
United States	-0.02		(0.07)	0.11	***	(0.03)	0.20	*	(0.08)	0.15	***	(0.02)	0.11	***	(0.02)	-1.11%	0.38	6.97%	0.00	-0.02	(0.07)	-0.02	(0.02)
**Average**	0.08		(0.04)	0.07	***	(0.02)	0.14	**	(0.05)	0.07	**	(0.02)	0.05	**	(0.02)	3.15%	0.12	5.98%	0.03	0.00	(0.05)	0.01	(0.02)

Controls: age, age^2, gender, employment, number of books at home, immigrant status, occupational classification of respondent's job, having children.

*** p<0.001; ** p<0.01; * p<0.05
