# Supplementary material for: Education and Self-Reported Health: Evidence from 23 Countries on the Role of Years of Schooling, Cognitive Skills and Social Capital
Source: PLoS One. 2016 Feb 22;11(2):e0149716. doi: 10.1371/journal.pone.0149716 (PMC4763098; doi:10.1371/journal.pone.0149716)
Supplement: S4 Table — (PDF) [file pone.0149716.s004.pdf]

**Table S4. Country level characteristics.**

| Country | ISO code | 2010 GDP per capita (current thousands US$) | 2010 Health expenditure, total (% of GDP) | Type of health system |
|---|---|---|---|---|
| Australia | AUS | 51.801 | 9.02292 | National health care systems |
| Austria | AUT | 46.593 | 11.1296 | Multiple insurers, with automatic affiliation systems |
| Canada | CAN | 47.464 | 11.1128 | National health care systems |
| Cyprus | CYP | 30.439 | 7.27646 | Not classified |
| Czech Republic | CZE | 19.76 | 7.4319 | Multiple insurers, with choice of insurer systems |
| Denmark | DNK | 57.648 | 11.0818 | National health care systems |
| England/N. Ireland (UK) | GBR | 38.362 | 9.37394 | National health care systems |
| Estonia | EST | 14.632 | 6.24878 | Not classified |
| Finland | FIN | 46.205 | 8.99042 | National health care systems |
| Flanders (Belgium) | BEL | 44.361 | 10.5577 | Multiple insurers, with automatic affiliation systems |
| France | FRA | 40.706 | 11.5543 | Multiple insurers, with automatic affiliation systems |
| Germany | DEU | 41.726 | 11.5567 | Multiple insurers, with choice of insurer systems |
| Ireland | IRL | 47.904 | 9.20766 | National health care systems |
| Italy | ITA | 35.878 | 9.40791 | National health care systems |
| Japan | JPN | 42.909 | 9.58899 | Multiple insurers, with automatic affiliation systems |
| Korea | KOR | 22.151 | 7.33434 | Single payer systems |
| Netherlands | NLD | 50.341 | 12.1468 | Multiple insurers, with choice of insurer systems |
| Norway | NOR | 87.646 | 9.42236 | National health care systems |
| Poland | POL | 12.530 | 7.02288 | Single payer systems |
| Slovak Republic | SVK | 16.510 | 8.50862 | Multiple insurers, with choice of insurer systems |
| Spain | ESP | 30.738 | 9.64808 | National health care systems |
| Sweden | SWE | 52.076 | 9.46877 | National health care systems |
| United States | USA | 48.374 | 17.0837 | Multiple insurers, with choice of insurer systems |

Data Source for GDP and Health expenditure: World Development Indicators, World Bank
Data Source for health system characteristics: OECD Health System Characteristics Survey 2012. April 2014 release.
